# Characterization of Liposomes for Cancer Cell Transfection

**DOI:** 10.2174/1874120700701010060

**Published:** 2007-10-26

**Authors:** Svetlana A Tatarkova, Satvinder Khaira

**Affiliations:** 1 Department of Physics, Durham University, South Road, Durham, DH1 3LE, UK; 2 University of Manchester, Manchester, M13 9PL, UK

## Abstract

We have characterized a broad range of liposome formulations with varying DcChol:DOPE ratio. Subsequent addition of DcChol to liposomes increases its positive surface charge. However, loading the nuclear acids did not neutralize the overall negative surface potential to a similar extent. The liposomes were tested by transfection of DNA in living cancer cells.

## INTRODUCTION

Highly efficient and low-toxic drug delivery system is a vital element of gene engineering technologies. One of most prominent candidates, capable of carrying a gene to the cells is a liposome, which can be designed specifically in a way that it’s chemical and physical properties would help it acquire a good affinity to the cells. Small in size (typically between 50 to 800 nm), sterically stabilized cationic liposomes allow remote loading of anionic nuclear acids by ion gradient [1, 2]. The performance of liposomes greatly relies on their chemical content. Although the delivery of liposomes is still held back by their toxicity and stability problems, when physical and chemical changes cause the chemical degradation and leakage of the encapsulated drug. So far the liposomes remain as the most popular non-viral vectors, that have many advantages over viral ones by being non-immunogenic, easy to produce and not oncogenic.

In this short Letter we discuss the use of single-molecule detection technique and wide-field light microscopy in order to fully assess the physico-chemical properties of liposomes and their transfection efficiency. For a full review of available microscopic techniques see [3, 4]. The different chemical formulations of liposomes were prepared with varying DcCholecterol: DOPE ratio. We examined their electrophoretic mobility in the capillary microchamber and determined an actual value of their electrostatic charge. The size of a liposome is conventionally estimated using light scattering methods or NMR. Our liposomes contained the fluorescent dye incorporated between lipid bilayers that help to detect them by their fluorescent emission. By observing the liposome diffusion in aqueous solution we determined their diffusion coefficient and hydrodynamic radius. The liposomes then were used to produce lipoplexes by mixing them with nuclear acids expressing GFP (Green Fluorescent Protein). Living cancer cells were transfected with these pre-designed lipoplexes and the expression level of GFP and diffusion were measured. We found that the diffusion rate and concentration of GFP was cell-type dependant with non-uniform distribution across the cell.

## MATERIALS AND METHODS

Fluorescence correlation spectroscopy (FCS) was performed on an up-right epi-fluorescent research microscope Nikon E800. We used an additional laser source (488 nm) and a home-built detection unit to carry out the spectroscopic measurements. More details of optical setting could be found in [5].

FCS is based on the detection of fluctuations of the fluorescence emitted by optically excited molecules. The fluctuations appear due to the Brownian motion of fluorescent molecules in and out of a volume illuminated by a tightly focused laser beam. The time during which a molecule passes through the illuminated volume is determined by its diffusion coefficient, so that the intensity fluctuations are nothing but the fluctuations of the number of fluorescent molecules presented in the illuminated volume. Once the time series of fluctuations are recorded the autocorrelation function is calculated in real time using computer-based data acquisition card ALV-5000 (ALV-GmbH, Langen, Germany). Later these experimental autocorrelation functions are transformed to yield the distribution function of the diffusion coefficients using ALV-software package.

**Fig. (1):**
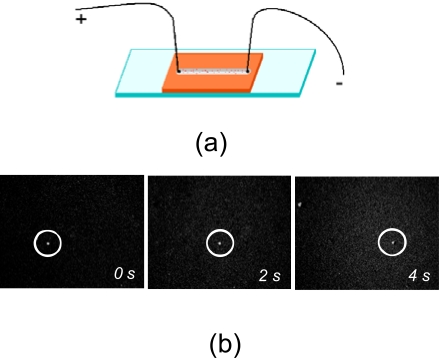
**a**. The sketch of the sample cell used for electrostatic charge measurement of liposomes. **b**. The microscopic fluorescent images of liposomes (circled) drifting under applied DC voltage.

For electrostatic charge measurements we combined the conventional wide-field light microscopy with time-lapse video-recording. The home-built sample cell filled with the liposome solution was used and a pair of point-like electrodes were attached to it as shown (Fig. **1a**). The sample cell was constructed by topping the microscopic glass with CoverWell perfusion chamber gasket (Molecular Probes), 4 mm wide, 1.0 mm deep and 32 mm long. The gasket had dual-access open ports so reagents could be quickly added and removed. These open ports were used also to insert the electrodes in the reagent solution. The DC voltage was applied across the cell chamber and was equal to +5 V. The fluorescent images of liposomes moving with a constant speed through the sample cell under the applied DC voltage were recorded using a CCD camera and the time-dependant position of the fluorescent molecule was subtracted from these images (Fig. **1b**). We used highly diluted liposome solutions to exclude liposome-liposome interaction and screening effects. Each measurement was repeated several times to extract an average velocity and its statistical deviation.

**Fig. (2):**
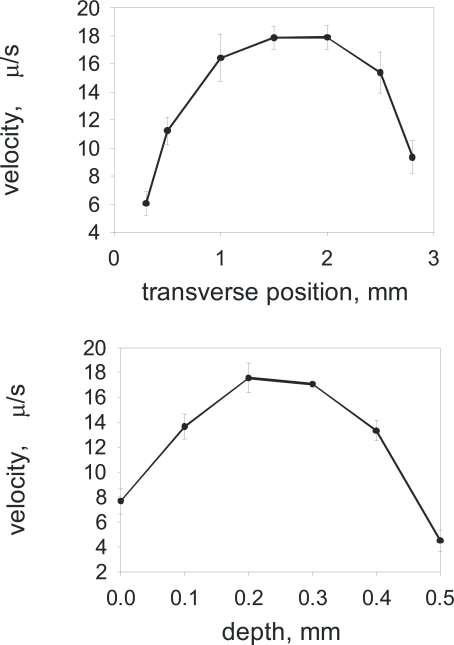
The calibration with fluorescent beads showing the variation of DC induced drift velocity across the sample cell - in width (on the top) and in depth (bottom).

We performed the preliminary calibration measurements using charged fluorescent beads in order to estimate the accuracy and variation of the electrophoretic velocities across the sample cell. The sample cell was filled with fluorescent beads (FluoSpheres® carboxylate-modified microspheres, 0.2 µm, yellow-green fluorescent (505/515)) and the relative velocities of the beads were measured as the beads were moving in the DC electric field while we focused our microscope objective to view the sample cell in a stepwise manner over its width (Fig. **2a**) and depth (Fig. **2b**). As we anticipated that the error of measurement could be very high, up to 50%, if the fluorescent bead is positioned off-centre of the sample cell. Therefore all electrostatic measurements of liposome charge were taken at the single fixed position, roughly at the centre of the sample chamber, recording only those liposomes which were in this focal plane away from the sample cell walls.

The liposomes were prepared by the pressing a plane lipid film with varying DcChol:DOPE ratio through a membrane with pores of diameter of 200 nm. Liposomes seal themselves to form a spherical shell. At the same time, a sufficiently large number of molecules of a fluorescent dye were packed between the lipid layers. The hydrophobic fluorophores DiIC_18_ (3) (Molecular Probes) were used with excitation at 549 nm and fluorescent emission at 565 nm.

The size of liposomes was measured by FCS (single molecule detection) and independently using conventional light scattering. In both cases the average diameter of liposomes did not exceed 173 nm (within an error bar).

Lipoplexes preparation was as follows: 0.25 ml liposomes in 500 μl solutions were mixed with 1 μg of DNA in 500 μl solution and left to develop for 15 minutes before performing the measurement of electrostatic charge. We routinely used FCS analysis in order to obtain an average hydrodynamic radius of lipoplexes. Average diameter was (275±13) nm for all formulations.

For the cell transfection experiment the cells of different cell lines (A549, KHT, HTGA, T470, GL) previously grown on the cover slip were washed in serum free media and then the lipoplexes were added into the cell. The cells were left for few hours for incubation (up to 5 hours) depending on the cell type. After that serum containing medium was added to fix the cells and after another 24 to 48 hours the cells with GFP expression were ready for measurement.

## ELECTROPHORETIC MOBILITY AND CHARGE MEASUREMENTS

First we have measured the electrophoretic mobility of liposome using 500 μm thick planar sample cell with water and liposomes suspended in it. When the voltage is applied in the plane of the cell, we monitor the motion of a selected liposome in the middle of the cell and measure the constant velocity of electrophoretic drift. This quick measurement allows us to determine the polarity of the carrier and a estimate the value of effective charge by measuring the migration speed of macromolecules in the solution.

We observed the particle electrophoretic migration on a monitor while performing image processing to resolve the particle migration speed, which then was presented in the electrons charge. As the velocities are relatively slow we can assume that the force of the applied electric field *F=q·E *is balanced by the Stokes drag force *F=6πηrv*,where *E* is effective electric field per unit length, *q* is an electrostatic charge, *r* is a liposome radius, *v* is a velocity and *η* is viscosity of aqueous solution. Therefore the charge of the liposome would be *q=6πηrv/E.*

The results are shown on Fig. (**3**). To add strength to our findings we have prepared separately two different formulations of anionic and one of neutral liposomes and tested them for electrophoretic mobility in the same experimental settings. The anionic liposomes (on the left) migrated in the opposite direction and are shown as being with a negative velocity. In this case two different anionic formulations PcChol:DOPE=x:1 were with subsequently added PcChol content that would normally give them bigger a charge. The neutral liposomes (shown as a point at the zero line) did not experience any drift; therefore their velocity is indicated at zero. Cationic liposomes as found smoothly increase their drifting velocity with increasing DcChol content. Exceptions were found for two formulations, DcChol:DOPE=4:1 and 2:1, which were moving unexpectedly fast indicating higher electrostatic charge. (There were some difficulties with their preparations as well, when a lot of lipid remained stacked to the membrane and was lost.)

Accordingly, the lipoplexes were differentiated by their migration velocities. It is clear that their charge drops after incorporating with liposomes to form a liposome-plasmid complex (lipoplexes). However we could not succeed in getting neutral final formulation of lipoplexes and they retained their negative charge. Nevertheless our measurements of their migration are very consistent with those previously measured for liposomes and this is a good indication of the good quality of our results. Migration drift proportionally slowed down as “more” cationic liposomes were deployed making them “nearly neutral”.

The data on Fig. (**3**) could be used to estimate the effective charge of liposomes/lipoplexes using the formula given above. The cationic liposome charge is ranging between (211±55) electrons for DcChol:DOPE=1:1 up to highest (549±37) electrons for the least formulation, DcChol:DOPE=10:1. For the lipoplexes we have the charge correspondingly in the range of (-475±35) electrons up to (-274±24) electrons. In physical chemistry it is common to use so-called Zeta-potential for these purposes. However, conventional Zeta-potential would give the value of electric, or zeta-potential, while it is more convenient to use the electron charge units.

## CELL TRANSFECTION

In the final stage we tested our pre-formulated liposomes for transfection efficiency. Reportedly the liposomes are used for the encapsulation of anti-cancer drugs and gene therapy and they are an efficient drug-delivery system. Recently they were used for encapsulation and targeting even with such sensitive substances as peptidic antimicrobials [6, 7]. Detailed mechanism of intermolecular interactions with cell membranes is very complex and recent studies stress to particular importance of dipole potential of membrane [8].

In the experiment of living cancer cells transfection with a vector expressing GFP we used the melanoma cell culture (KHT mice line) and lipoplexes prepared as described above. The solution of lipoplexes were added into a nutrient medium DMEM and remained there for 2 hours for incubation. Then, the cells were washed twice with a physiological solution to remove the remains of liposomes and viewed under the microscope. Overall picture of cell transfection (KHT cell line, transfected with liposomes DcChol:DOPE=3:2) and taken with ×4 objective is shown on Fig. (**4a**). On Fig. (**4b**) a group of cells is shown with greater magnification (×40).

The conventional way to count fluorescently labeled cells (in our case with GFP) is using the fluorescence-activated cell sorter (FACS-machine). The sorter funnels cells through a tiny channel and counts the flashes as labeled cells are illuminated by a laser. After a visual examination under the microscope we found that the overall level of GFP expression was relatively low. These observations were consistent with those received using the cell sorter. We received about 40 counts from the sample containing cells with GFP expression, while in comparison the control sample of wild cells gave the baseline between 1 to 10 counts.

FCS might be used as an alternative way to measure the GFP expression inside the cell. While more sophisticated statistical data over entire population of cells are provided by the cell-sorter, detailed in-cell fluorescence count could be measured with point-like measurement by FCS [8-10]. That is, assuming that the fluorescent protein molecules are freely distributed inside the cell and undergo Brownian motion restricted only by the cell boundaries. Tight focused laser light illuminates a small area inside the cell and detects fluctuations of the number of proteins molecules within this illuminated area. GFP reportedly sited for its high quantum yield of fluorescence and 488 nm wavelength of an argon laser perfectly serves for fluorescence excitation. At relatively moderate laser power the protein fluorescence is stable and no bleaching is observed during the measurement.

Although the expression level was relatively low (as supported by the fluorescence counts measurement with FACS machine) we could still measure the average concentration of GFP with FCS. From Fig. (**4a-b**). we can see that GFP expression appears in the number of cells. At the same time the protein itself is distributed non-uniformly across the cell leaving bright and dark spots of green fluorescence that is reflected in FCS data. In fact, in most cases, we had quite a few protein molecules in the excitation volume. Summarizing all measurements and normalizing to the detection volume we found total concentration of GFP did not exceed few a nM. We did not find significant difference in the level of expression between different cell types, although we did not pursue further systematic studies of this effect. In previous studies it was found [8, 11] that the physical properties of liposome formulation and their biological activity do not always correlate, and more systematic studies are needed to assess the effect of vehicle formulation on their biological activity using routine *in vitro* cell transfection.

## CONCLUSIONS

We have successfully tested the wide range of liposome formulations and their complexes with vector expressing GFP in gene therapy applications to living cancer cells. FCS is proved to be useful tool to assess a sparse concentration of fluorescent drug in cells and biological tissue [9, 10, 12]. Although we could not establish a direct link between chemical formulation of lipid vehicles and their transfection efficiency we have demonstrated the variety of techniques, which are easy to assemble on an epi-fluorescent microscope stage and easy to use for full physico-chemical characterization of drug delivery systems.

## Figures and Tables

**Fig. (3):**
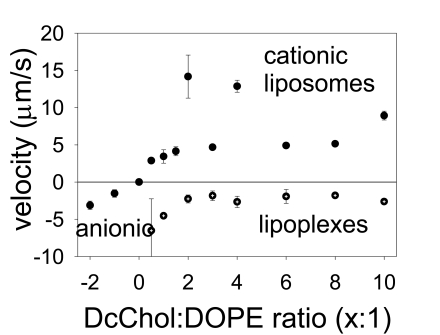
The velocities of liposome electrophoretic migration in DC static field are proportional to their electrostatic charge.

**Fig. (4):**
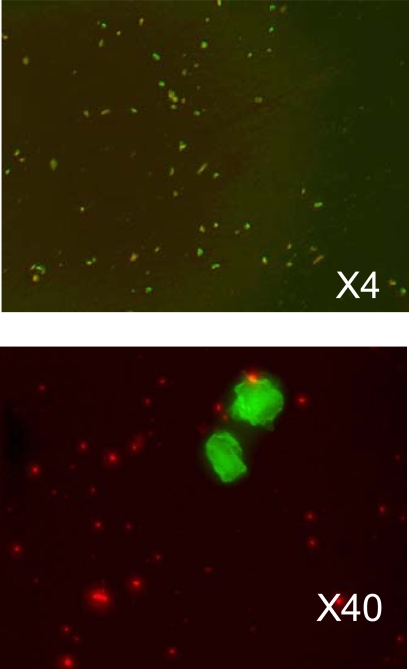
The microscopic images of transfected cancer cells. The upper image is taken with 4-times magnification and show the overall density of the transfected cells. The lower image shows transfected cells in more detail. Green fluorescence is from GFP, and red fluorescence - from liposomes.
